# Supplementary material for: Nanoscale electrical conductivity imaging using a nitrogen-vacancy center in diamond
Source: Nat Commun. 2018 Jun 19;9:2406. doi: 10.1038/s41467-018-04798-1 (PMC6008463; doi:10.1038/s41467-018-04798-1)
Supplement: Supplementary file 1 — Supplementary Information [file 41467_2018_4798_MOESM1_ESM.pdf]

Supplementary Information
Nanoscale electrical conductivity imaging using a nitrogen-vacancy center in diamond

Ariyaratne *et al.*

SUPPLEMENTARY NOTE 1: MAGNETIC SPECTRAL DENSITY EMANATED BY A THIN CONDUCTING FILM

To quantitatively measure a material's conductivity using an NV center in diamond, we use theory developed by [1, 2] to describe the magnetic Johnson noise produced by an infinite conducting slab of finite thickness a. The experiments presented here probe polycrystalline metals at room temperature, and therefore we do not consider non-local effects such as those observed in [3]. We then develop an analytic solution to the magnetic spectral density from a conductor with finite thickness a. For consistency with referenced works, in this supplement we define the metal thickness as a and the distance of the NV to the conductor surface as z, instead of t_{film} and d, as used in the main text, respectively.

First, consider the commonly presented solution as in [1, 3] for the magnetic spectral density emanated by a metallic half-space

$$\begin{aligned} S^{z'}_{B,\text{half-space}} &= \frac{\mu_0^2 k_B T\sigma}{16\pi}\left(\frac{1}{z}\right) \\ S^{x'}_{B,\text{half-space}} &= S^{y'}_{B,\text{half-space}} = \frac{S^{z'}_{B,\text{half-space}}}{2} \end{aligned} \tag{1}$$

Where μ_0 is the vacuum permeability, k_{B} the Boltzmann constant, T the temperature, and σ the metal conductivity. A half-space a distance z away is equivalent to the summation of a slab of thickness a a distance z away and a half-space a distance $z+a$ away.

$$\begin{aligned} S^{z'}_{B,\text{half-space}}(z) &= S^{z'}_{B,\text{slab}}(z) + S^{z'+a}_{B,\text{half-space}}(z+a) \\ S^{z'}_{B,\text{slab}}(z) &= S^{z'}_{B,\text{half-space}}(z) - S^{z'+a}_{B,\text{half-space}}(z+a) \\ S^{z'}_{B,\text{slab}} &= \frac{\mu_0^2 k_B T\sigma}{16\pi}\left(\frac{1}{z} - \frac{1}{z+a}\right) \end{aligned} \tag{2}$$

This elegant solution neglects the effect of the boundary at $z+a$. To confirm its validity, we rigorously derive the same result using Fresnel coefficients for a finite-thickness film.

Derived using the fluctuation-dissipation theorem and a magnetic Green's tensor, the magnetic spectral density tensor at angular frequency ω and temperature T can be expressed as

$$S_B^{ij} = \frac{\hbar\omega^3}{4\pi\epsilon_0 c^5}\coth\left(\frac{\hbar\omega}{2k_B T}\right)s_{ij} \tag{3}$$

Where $\hbar\omega^3/4\pi\epsilon_0 c^5$ has units of magnetic spectral density (Tesla^2 Hz^{-1}), and s_{ij} is a dimensionless tensor. For simplicity we consider a coordinate system x', y', z' with the z'-axis perpendicular to the metal, and z being the distance to the nearest face of the metal film, such that s_{ij} becomes a diagonal tensor with elements

$$\begin{aligned} s_{x'x'} = s_{y'y'} &= \frac{1}{2}\text{Re}\int_0^\infty du\,\frac{u\left(r_p(u) + (u^2-1)r_s(u)\right)}{\eta}e^{2izk\eta} \\ s_{z'z'} &= 1\,\text{Re}\int_0^\infty du\,\frac{u^3 r_s(u)}{\eta}e^{2izk\eta} \end{aligned} \tag{4}$$

$$\eta(u) = \begin{cases} \sqrt{1-u^2} & 0 \le u \le 1 \\ i\sqrt{u^2-1} & u > 1 \end{cases} \tag{5}$$

Where we use $k = |\omega|/c$ and Fresnel coefficients r_p and r_s. For simplicity we drop the double index on the diagonal elements such that $s_{z'} \equiv s_{z'z'}$ As shown in [2], for a metal of finite thickness a these Fresnel coefficients are

$$r_s(u) = \frac{k_1^2 - k_2^2}{k_1^2 + k_2^2 + 2ik_1k_2\cot(k_2 a)} \qquad r_p(u) = \frac{(\epsilon_2 k_1)^2 - (\epsilon_1 k_2)^2}{(\epsilon_2 k_1)^2 + (\epsilon_1 k_2)^2 + 2i\epsilon_1\epsilon_2 k_1 k_2\cot(k_2 a)} \tag{6}$$

$$k_1 \equiv k\sqrt{\epsilon_1 - u^2} \qquad\qquad k_2 \equiv k\sqrt{\epsilon_2 - u^2} \tag{7}$$

Where $\epsilon_{1,2}(\omega)$ are the relative dielectric functions of the two media, which in this case are $\epsilon_1 \equiv \epsilon_{\text{vacuum}} = 1$ and $\epsilon_2 \equiv \epsilon_{\text{metal}}$. In the frequency regime of the NV level splitting $\omega \approx 2\pi*$ 2.8 GHz, and for metal conductivity σ

$$\epsilon_2 \approx \frac{i\sigma}{\epsilon_0\omega} \tag{8}$$

Thus, the above expressions relate the magnetic noise spectral density at the NV produced by a metal with conductivity σ.

As described in [1] the integrals are dominated by values of $u \approx 1/(2kz) \approx 10^6$ for our experiment where $z \approx 10$ nm and $\omega \approx 2\pi * 2.8$ GHz. For the metallic conductivities studied here, $|\epsilon_2|^{1/2} \approx 10^3$ - 10^4, and thus $u \gg |\epsilon_2|^{1/2}$. This is equivalent to stating that the skin depth $\delta \gg z$, where $|\epsilon_2|^{1/2} = \sqrt{2}/k\delta$. We now make the critical approximation that $\sqrt{\epsilon - u^2} \approx \sqrt{1 - u^2} \approx iu$ and set $\epsilon_1 = 1$ for vacuum

$$r_s(u) = \frac{1 - u^2 - \epsilon_2 + u^2}{1 - u^2 + \epsilon_2 - u^2 + 2i\sqrt{1-u^2}\sqrt{\epsilon_2 - u^2}\cot\left(ka\sqrt{\epsilon_2 - u^2}\right)} = \frac{1 - \epsilon_2}{1 + \epsilon_2 - 2u^2(1 + \coth(kau))} \tag{9}$$

Multiplying the numerator and denominator by the complex conjugate of the denominator

$$r_s(u) = \frac{(1 - \epsilon_2)\left(-\epsilon_2 - 2u^2(1 + \coth(kau))\right)}{|\epsilon_2|^2 + 4u^4(1 + \coth(kau))^2} \tag{10}$$

Applying $\sqrt{1 - u^2} \approx iu$ to the $s_{z'}$ integral in Supplementary Eq. 4 yields

$$\begin{aligned} s_{z'} &= 1\,\text{Re}\int_0^\infty du\,\frac{u^3}{iu} r_s(u) e^{2izk(iu)} \\ &= 1\,\text{Re}\int_0^\infty du\,\frac{u^2}{i}\frac{(1 - \epsilon_2)\left(-\epsilon_2 - 2u^2(1 + \coth(kau))\right)}{|\epsilon_2|^2 + 4u^4(1 + \coth(kau))^2} e^{-2kzu} \end{aligned} \tag{11}$$

$\coth(kau) + 1$ is a real, positive number always greater than 2, thus $4u^4(1 + \coth(kau))^2 \gg |\epsilon_2|^2$. Taking the real part,

$$s_{z'} = \frac{\epsilon_2}{i}\int_0^\infty du \frac{2u^4(1 + \coth(kau))}{4u^4\left(1 + \coth(kau)\right)^2} e^{-2kzu} \tag{12}$$

Noting that $1 + \coth(kau) = 2e^{kau}/(e^{kau} - e^{-kau})$, and substituting in $\epsilon_2 = i\sigma/(\epsilon_0\omega)$

$$\begin{aligned} s_{z'} &= \frac{\sigma}{\epsilon_0\omega}\int_0^\infty du \frac{(e^{-2kzu})(e^{kau} - e^{-kau})}{4e^{kau}} \\ &= \frac{\sigma c}{8\epsilon_0\omega^2}\left(\frac{1}{z} - \frac{1}{z + a}\right) \end{aligned} \tag{13}$$

Using the same approximations as above one finds $r_p(u) \approx 1$, and then that $s_{x'} = s_{y'} \approx s_{z'}/2$. Further, at room temperature and $\omega \approx 2\pi*$ 2.8 GHz, $\coth(\hbar\omega/2k_BT) \approx 2k_BT/\hbar\omega$. Calculation of the dimensionless tensor s_{ij} then yields the magnetic spectral density tensor elements

$$\begin{aligned} S_B^{z'} &= \frac{\hbar\omega^3}{4\pi\epsilon_0 c^5}\frac{2k_BT}{\hbar\omega}\frac{\sigma c}{8\epsilon_0\omega^2}\left(\frac{1}{z} - \frac{1}{z + a}\right) \\ S_B^{z'} &= \frac{\mu_0^2 k_B T\sigma}{16\pi}\left(\frac{1}{z} - \frac{1}{z + a}\right) \\ S_B^{x'} &= S_B^{y'} = \frac{S_B^{z'}}{2} \end{aligned} \tag{14}$$

As expected, Supplementary Eq. 14 simplifies to the half-space solution as one takes film thickness $a \to \infty$, and the calculation performed here matches our geometric derivation in Supplementary Eq. 2. Importantly, the Johnson noise is white for the relevant GHz frequencies.

We now complete this analysis by relating the magnetic spectral density to the ensuing relaxation rate from $|m_s = 0\rangle$ to $|m_s = -1\rangle$ for a spin-1 system like the NV.

SUPPLEMENTARY NOTE 2: POPULATION DYNAMICS OF THE NV THREE-LEVEL SYSTEM

As described in [4], the relaxation rate of $|m_s = 0\rangle$ to $|m_s = 1\rangle$ is approximately the same as the relaxation rate of $|m_s = 0\rangle$ to $|m_s = -1\rangle$. Denoting this rate as Ω, and denoting the double-quantum transition rate between $|m_s = 1\rangle$ and $|m_s = -1\rangle$ as γ, the population dynamics are thus described by

$$\frac{d}{dt}\begin{pmatrix}\rho_0\\ \rho_{-1}\\ \rho_1\end{pmatrix} = \begin{pmatrix}-2\Omega & \Omega & \Omega\\ \Omega & -\Omega-\gamma & \gamma\\ \Omega & \gamma & -\Omega-\gamma\end{pmatrix}\begin{pmatrix}\rho_0\\ \rho_{-1}\\ \rho_1\end{pmatrix} \tag{15}$$

Solving the eigenvalue-eigenvector problem, we see that

$$\begin{pmatrix}\rho_0\\ \rho_{-1}\\ \rho_1\end{pmatrix} = C_1\begin{pmatrix}2\\ -1\\ -1\end{pmatrix}e^{-3\Omega t} + C_2\begin{pmatrix}0\\ 1\\ -1\end{pmatrix}e^{-(\Omega+2\gamma)t} + C_3\begin{pmatrix}1\\ 1\\ 1\end{pmatrix} \tag{16}$$

As described in the main text, we optically polarize the NV into $|m_s = 0\rangle$. Ideally $\rho_0(0) = 1$ and $\rho_{-1}(0) = \rho_1(0) = 0$, but we account for imperfect optical polarization by defining η such that $\rho_0(0) = 1 - 2\eta$ and $\rho_{-1}(0) = \rho_1(0) = \eta$, where η is nominally 0.05 [5]. With these initial conditions, we see that

$$\rho_0(t) = \frac{1}{3} + 2\left(\frac{1}{3} - \eta\right)e^{-3\Omega t} \tag{17}$$

$$\rho_{-1}(t) = \rho_1(t) = \frac{1}{3} - 1\left(\frac{1}{3} - \eta\right)e^{-3\Omega t} \tag{18}$$

In a spin-dependent photoluminescence (SDPL) measurement sequence, as shown in Supplementary Figure 3a, optical polarization is followed by evolution for a dark time τ, and then the NV photoluminescence (PL) signal S is measured, where S is given by

$$S = A\rho_0 + B\rho_{-1} + B\rho_1 + \text{Background} \tag{19}$$

A and B describe the brightness of the $|0\rangle$ and $|\pm1\rangle$ states, respectively. The $|-1\rangle$ and $|1\rangle$ states are taken to be equally bright. Background designates any non-NV^- signal that may come from *e.g.* APD dark counts, light leakage, and any population in the neutral NV^0 due to imperfect charge state polarization. We then repeat this experiment, but immediately before readout a resonant microwave pulse swaps the population of the $|-1\rangle$ and $|0\rangle$ states. This second measurements yields the signal S_{swap}:

$$S_{\text{swap}} = B\rho_0 + A\rho_{-1} + B\rho_1 + \text{Background} \tag{20}$$

We then take the difference between the two measurements S and S_{swap} to calculate the quantity S_{diff}

$$S_{\text{diff}} = S - S_{\text{swap}} = (A - B)(1 - 3\eta)\, e^{-3\Omega t} = Ce^{-3\Omega t} \tag{21}$$

where we include the imperfect spin polarization in the definition of contrast C. In Fig. 1c of the main text we plot a normalized version of S_{diff}, where the contrast is normalized to 1 by means of reference measurements of $S_{\text{diff}}(0)$. We fit to an exponential decay with decay rate

$$\frac{1}{T_1} = \Gamma = 3\Omega \tag{22}$$

Double-quantum relaxation, imperfect spin polarization, and any background signal do not modify the measured Γ. However, AOM leakage during long, ms-scale dark times, can repolarize the NV at a rate δ, which changes the form of the decay curve to $S_{\text{diff}} = C\exp\left(-\left(3\Omega+\delta\right)t\right)+y_0$. y_0 is an inevitable offset since the dark, steady-state population with a slow polarization rate will not be an even thermal mixture of the 3 spin states. We find that several nW of laser leakage can repolarize the NV at a rate of ~ 50 Hz. Thus we take care to reduce laser leakage to below 1 nW and confirm that $S_{\text{diff}} \to 0$ as $t \to \infty$.

SUPPLEMENTARY NOTE 3: NV RELAXATION RATE NEAR A CONDUCTOR

We now calculate Ω_{metal} via perturbation theory applied to the NV Hamiltonian, with NV coordinates x, y, z. The NV's magnetic moment axis, the z-axis, makes an angle θ with the z'-axis. We can further select this axis to lay entirely in the x'-z' plane without any loss of generality, since the x' and y' components of the magnetic spectral density tensor are equivalent.

Hence, $B_x = \cos(\theta)B_{x'} + \sin(\theta)B_{z'}$ and $B_y = B_{y'}$. Note that the x', y', z' components of the magnetic field are all uncorrelated, and thus $S_B^x = \cos^2(\theta)S_B^{x'} + \sin^2(\theta)S_B^{z'}$ and $S_B^y = S_B^{y'}$. We use the formalism described in [1] to describe the relaxation rate under the interaction Hamiltonian H', as described in the main text with NV gyromagnetic ratio γ and raising and lowering operators S_+ and S_-

$$H' = \gamma B_z S_z + \frac{1}{2}\gamma(B_x - iB_y)S_+ + \frac{1}{2}\gamma(B_x + iB_y)S_- \tag{23}$$

$$\Omega_{0\to-1,\text{metal}} = |\langle 0|\gamma S_z|-1\rangle|^2 S_B^z + |\langle 0|\frac{1}{2}\gamma S_+|-1\rangle|^2\left(S_B^x + S_B^y\right) + |\langle 0|\frac{1}{2}\gamma S_-|-1\rangle|^2\left(S_B^x + S_B^y\right) \tag{24}$$

$$\Gamma_{\text{metal}} = 3\Omega_{\text{metal}} = 3\gamma^2\left(\frac{1}{2}\cos^2(\theta)S_B^{x'} + \frac{1}{2}S_B^{y'} + \frac{1}{2}\sin^2(\theta)S_B^{z'}\right) \tag{25}$$

The diamonds used in this work are (100) oriented, and thus all four possible NV orientations make an angle $\theta = \arccos(\sqrt{1/3}) \approx 54.7^\circ$ with the z'-axis, making our analysis independent of the NV orientation.

We now finish our derivation of relaxation rate by using our derived expression for the magnetic spectral density tensor.

$$\Gamma_{\text{metal}} = \gamma^2\frac{3\mu_0^2 k_B T\sigma}{32\pi}\left(1 + \frac{1}{2}\sin^2(\theta)\right)\left(\frac{1}{z} - \frac{1}{z+a}\right) \tag{26}$$

$$\Gamma_{\text{metal}} = \gamma^2\frac{\mu_0^2 k_B T\sigma}{8\pi}\left(\frac{1}{z} - \frac{1}{z+a}\right) \tag{27}$$

Note that in the main text we define $d \equiv z$ and $t_{\text{film}} \equiv a$.

SUPPLEMENTARY NOTE 4: MAGNETIC SPECTRAL DENSITY EMANATED BY A FINITE-GEOMETRY CONDUCTOR

The magnetic fluctuations emanating from a conductor can be derived in an alternate method to that presented in Supplementary Note 1.This method is the one presented in the main text and it offers much physical intuition. However, we warn that the results for the x' and y' components of the magnetic spectral density are overestimated by a factor of 3 because we do not account for boundary conditions at the surface. Nevertheless, this method has further utility as it allows us to estimate the magnetic noise from finite geometries [6].

In the Drude model, the ith component ($i = x', y', z'$) of an electron's velocity v_i will be correlated in time by $\langle v_i(t)v_i(t+t')\rangle = v_i^2 * \exp(-t/\tau_c)$, where τ_c is the mean electron collision time. With the Wiener-Khinchin theorem, taking a Fourier transform of this velocity autocorrelation function yields a two-sided velocity spectral density

$$S_v^i(\omega) = v_i^2\frac{\tau_c}{1+\omega^2\tau_c^2} \tag{28}$$

The velocity noise produced by an electron with mass m and mean thermal energy $3k_{\rm B}T/2$ is thus

$$\overline{S_v^i}(\omega) = \frac{2k_{\rm B}T}{m}\frac{\tau_c}{1+\omega^2\tau_c^2} \tag{29}$$

The Biot-Savart law gives the magnetic field from a moving electron as $B_{z'}(t) = \mu_0(v_{y'}(t)x' - v_{x'}(t)y')e/4\pi r'^3$, where e is the electronic charge and r' is the distance to the electron. From the autocorrelation function $\langle B_{z'}(t)B_{z'}(t+t')\rangle$ we calculate $S^{z'}_{B,\text{one electron}} = \mu_0^2 e^2(y'^2 S_v^{x'} + x'^2 S_v^{y'})/(4\pi r'^3)^2$. Note that using the Wiener-Khinchin theorem to find the velocity spectral density is equivalent to the argument in the main text where we start at the Johnson-Nyquist expression for current spectral density.

We assume all of the electrons in the metal are uncorrelated, and thus their magnetic field spectral densities add incoherently. Summing up the mean electron contributions from volume elements with electron density n, we then calculate $S_B^{z'}$ from an infinite film of thickness a a distance z away,

$$S_B^{z'} = \frac{\mu_0^2 k_{\rm B}T}{16\pi}\left(\frac{ne^2}{m}\frac{\tau_c}{1+\omega^2\tau_c^2}\right)\int_{V'} dV' \frac{4\left(x'^2+y'^2\right)}{2\pi r'^6} \tag{30}$$

$$S_B^{z'} = \frac{\mu_0^2 k_{\rm B}T}{16\pi}\sigma \int_z^{z+a}\int_0^{\infty} \frac{4\rho'^3 d\rho' dz'}{(\rho'^2+z'^2)^3} \tag{31}$$

$$S_B^{z'} = \frac{\mu_0^2 k_{\rm B}T\sigma}{16\pi}\left(\frac{1}{z} - \frac{1}{z+a}\right) \tag{32}$$

where σ is the electrical conductivity with $\sigma \equiv \mathrm{Re}\left[\sigma(\omega)\right] \approx \sigma(0)$ for $\omega \sim 2\pi * 2.87$ GHz. This calculation method yields the correct expression for $S_B^{z'}$, which was rigorously derived in Supplementary Note 1 using the fluctuation-dissipation theorem. However, because we did not account for boundary conditions at the surface $S_B^{x'}$ and $S_B^{y'}$ would be overstated by a factor of 3 with this method of calculation [6].

In Fig. 2 of the main text we study the noise from plateau tips of 1.5 μm radius, which we approximate to be infinite films. We can conveniently estimate the deviation from the infinite-film approximation by performing the radial integral in Supplementary Eq. 31 to 1.5 μm, instead of ∞. Similarly, we calculate integral expressions for $S_B^{x'}$ and $S_B^{y'}$ and integrate to 1.5 μm. We then compare $\Gamma_{\text{Biot},\infty}$ to $\Gamma_{\text{Biot},1.5\mu\text{m}}$. Although the simplistic Biot-Savart calculation ignores surfaces and thus is slightly skewed in magnitude, by considering the volume integral we closely estimate the relative deviation from the infinite-film model. We find that this relative deviation is approximately 10% of the experimental error for Γ_{metal} for Fig. 2, which we deem to be negligible for this study.

This Biot-Savart method of calculation also allows us to form the basis for the simulation in Fig. 4 of the main text. We employ a Monte Carlo simulation of a single electron in a metal, which has previously been developed to estimate the velocity autocorrelation function and spectral density inside finite geometries [7]. We simulate an electron with a given velocity and a certain probability to scatter dictated by the bulk, mean collision time τ_c. The electron also scatters at boundaries, however, and this effect modifies the velocity spectral density: near the boundary the effective collision time τ_c will decrease, which stretches the velocity spectral density (Supplementary Eq. 28) and suppresses the magnitude of the noise at $\omega \sim 2\pi * 2.87$ GHz. For the simulation in Fig. 4 of the main text, we perform a Monte Carlo simulation of the electron trajectory, but instead of calculating the velocity noise we use the Biot-Savart law to explicitly calculate the magnetic autocorrelation spectral density for every point in free space. This simulation allows us to account for the finite size of the conductor as well as the approximate noise suppression at boundaries. Since the noise from two conductors will be uncorrelated, we can sum the spectral densities from the two conducting blocks in Fig. 4. This can be done for any geometry. In order to ensure that the magnitude of the magnetic spectral density is properly estimated, we employ the same logic as for the estimation described above: we compare the simulated $\Gamma_{\text{sim, finite}}$ to $\Gamma_{\text{sim},\infty}$ to estimate the relative deviation from an infinite film and we then apply Supplementary Eq. 14 to obtain the absolute magnitude.

SUPPLEMENTARY NOTE 5: TEMPERATURE CONTROL AND SPM STABILITY

Temperature fluctuations play an important role in our system's stability; a 1 mK change results in $\sim$ 1 nm of drift of the SPM cantilever relative to the NV, as a consequence of using materials with different coefficients of thermal expansion. To mitigate thermal drifts, we implement several layers of thermal isolation and active temperature feedback. Our experimental apparatus is enclosed in an insulating box on an optical table, with the entire optical table isolated by curtains. The laboratory temperature is stabilized to within 1K. A PID loop measures voltage across a thermistor with a sensitivity of 0.1 mK Hz$^{-1/2}$ and then heats thin resistance wire in feedback control. Thin

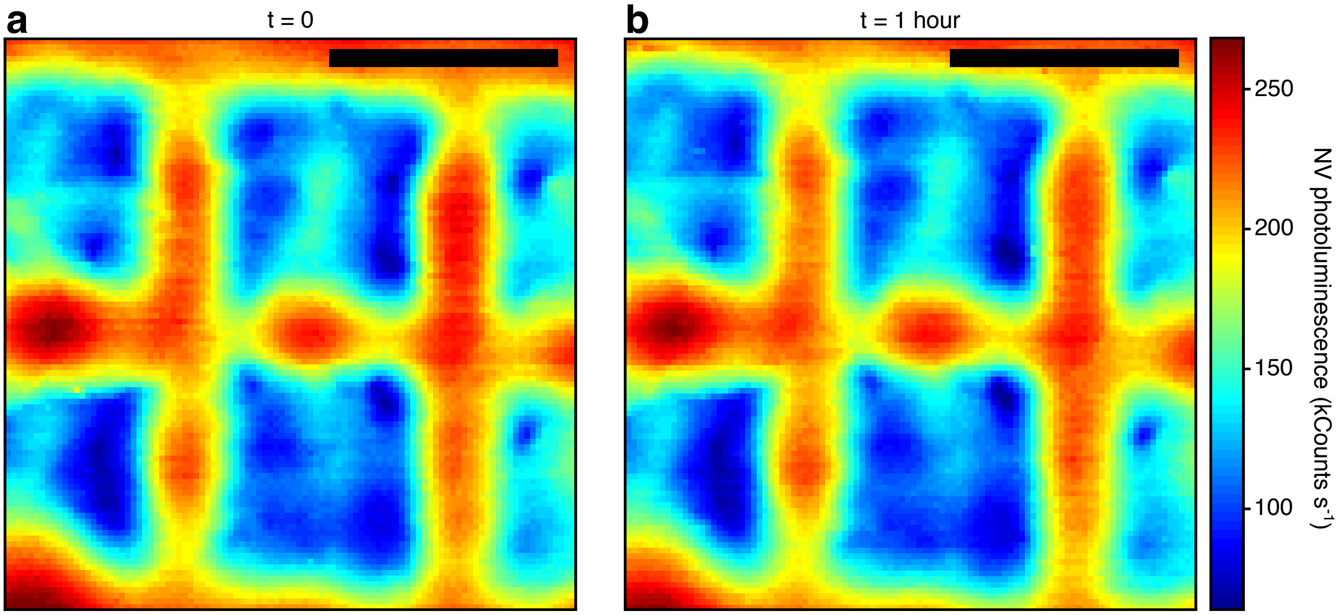

Supplementary Figure 1: NV-photoluminescence (PL)-based image registration for NV-sample drift correction. **a** and **b** are subsequent PL scans, taken 1 hour apart, of the nanopatterned sample in Fig. 3 of the main text. An image registration algorithm calculates an (x, y) shift of $(-0.5, 4.5)$ nm. Scale bars are 400 nm. The spatial PL modulation results from several factors that depend on the NV's position relative to the tip: the Purcell effect, a change in the NV's dielectric environment, and an interferometric effect between the incoming excitation laser and the tip-reflected laser [10]. The large PL modulation is consistent with small (∼10-20 nm) NV-sample separation.

resistance wire is optimal, with a small thermal mass and large surface area relative to its volume, yielding responsive feedback. This also allows us to distribute the heating sources evenly around the insulating box, which is critical in minimizing temperature gradients.

We achieve temperature stability to within 1 mK on the timescale of weeks. However, ambient changes in temperature outside the box can cause the temperature control system to introduce changing temperature gradients inside the box, which are the present limiting factor for drift.

In order to correct for these drifts, we employ image registration every 1-2 hours, using either a topographic AFM image or an NV PL image (the one with the sharper features is chosen for image registration). Two example PL images taken an hour apart in time are shown in Supplementary Figure 1. We use an image registration algorithm that allows for subpixel correction to ~ 1 nm precision [8, 9].

SUPPLEMENTARY NOTE 6: OPTIMAL T_1 MEASUREMENT

For the exponentially decaying signal $S_{\text{diff}} = C\exp(-t/T_1)$, the most time-efficient method of measuring T_1 is by acquiring data at $\tau = 0$ and $\tau \approx 0.7\ T_1$, which we show here.

Consider a weighted, least-squares linear fit for data with relationship $y_i = A + Bx_i$ with weights $w_i = 1/\sigma_{y_i}^2$, where σ_{y_i} is the standard deviation of the measured y_i values. One can calculate, then, that the variance on the fitted value of B is

$$\sigma_B^2 = \frac{\sum w_i}{\sum w_i \sum w_i x_i^2 - \left(\sum w_i x_i\right)^2} \tag{33}$$

If we linearize our signal S_{diff}, referred to now as S for brevity, we find that

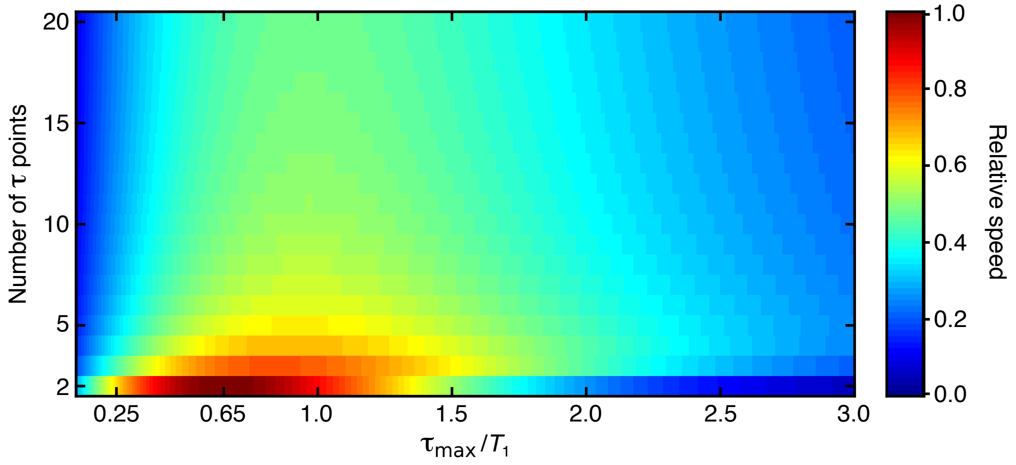

Supplementary Figure 2: T_1 measurement speed with different numbers of τ points spaced from $\tau = 0$ to $\tau = \tau_{\text{max}}$. The optimal measurement sequence is a measurement at $\tau = 0$ and at $\tau = 0.65\ T_1$. We assume a per-shot overhead time of $0.01\ T_1$. Deviating from $\tau \approx 0.7\ T_1$ greatly increases measurement time, stressing the necessity of adaptive-τ measurements for imaging.

$$\ln(S_i) = \ln(C) - \Gamma\tau_i \tag{34}$$

$$\sigma_{\ln(S_i)} = \frac{\sigma_S}{S_i} \tag{35}$$

$$\sigma_\Gamma^2 = \frac{\sigma_S^2 \sum S_i^2}{\sum S_i^2 \sum S_i^2 \tau_i^2 - \left(\sum S_i^2 \tau_i\right)^2} \tag{36}$$

We consider $\sigma_S^2 = \sigma_1^2/N$, where N is the number of repetitions and σ_1^2 is the per-shot measurement error, which we assert is the same for each τ_i. Consider a per-shot overhead time t_{extra} and dark times τ_i such that $N \sum(\tau_i + t_{\text{extra}}) = T$, with T the total measurement time. Thus, one finds that

$$\sigma_\Gamma^2 = \frac{\sigma_1^2}{T} \frac{\sum e^{-2\Gamma\tau_i} \sum(\tau_i + t_{\text{extra}})}{\sum e^{-2\Gamma\tau_i} \sum \tau_i^2 e^{-2\Gamma\tau_i} - \left(\sum \tau_i e^{-2\Gamma\tau_i}\right)^2} \tag{37}$$

In Supplementary Figure 2 we plot the relative speed $(1/\sigma_\Gamma^2)$ for N linearly spaced τ values between $\tau = 0$ to τ_{max}, as a function of N and τ_{max}. The plot shows a clear maximum in measurement speed at $\tau \approx 0.7\ T_1$. Supplementary Figure 2 thus emphasizes the importance of an adaptive-τ algorithm when imaging over an area where T_1 varies. For example, in the area imaged in Fig. 3 of the main text T_1 varies from 0.5 to 5 ms.

SUPPLEMENTARY NOTE 7: SPIN-TO-CHARGE READOUT ON A SHALLOW NV CENTER

For Fig. 3c of the main text we implement spin-to-charge conversion (SCC) readout [5] on a shallow NV center with a measurement sensitivity 5x the spin projection noise (SPN) limit. The principle behind SCC readout is selectively ionizing the $|m_s = 0\rangle$ spin state into the neutral charge state (NV^0) while leaving the $|m_s = \pm 1\rangle$ spin state in the negative charge state (NV^-). The charge state is then readout with a yellow (594 nm) laser that selectively excites only the NV^- charge state. Supplementary Figure 3b and Supplementary Figure 3c illustrate the SCC measurement scheme and show an example SCC T_1 measurement that is 5x the SPN limit.

The SPN limit is the result of a Bernoulli distribution: each projective measurement will result in a success $|m_s = 0\rangle$ or a failure $|m_s = \pm 1\rangle$. We make a measurement at $\tau \approx 0.7\ T_1$, for which the spin population is roughly split in half between $|m_s = 0\rangle$ and $|m_s = \pm 1\rangle$. If the population is split in half between the two outcomes, this results in a standard deviation of 1/2. Since each measurement of S_{diff} is the difference of two measurements (S and S_{swap}), whose errors add in quadrature, the SPN-limited standard error σ_{SPN} of N measurements of S_{diff} is

$$\sigma_{\text{SPN}} = \frac{1}{\sqrt{2N}} \tag{38}$$

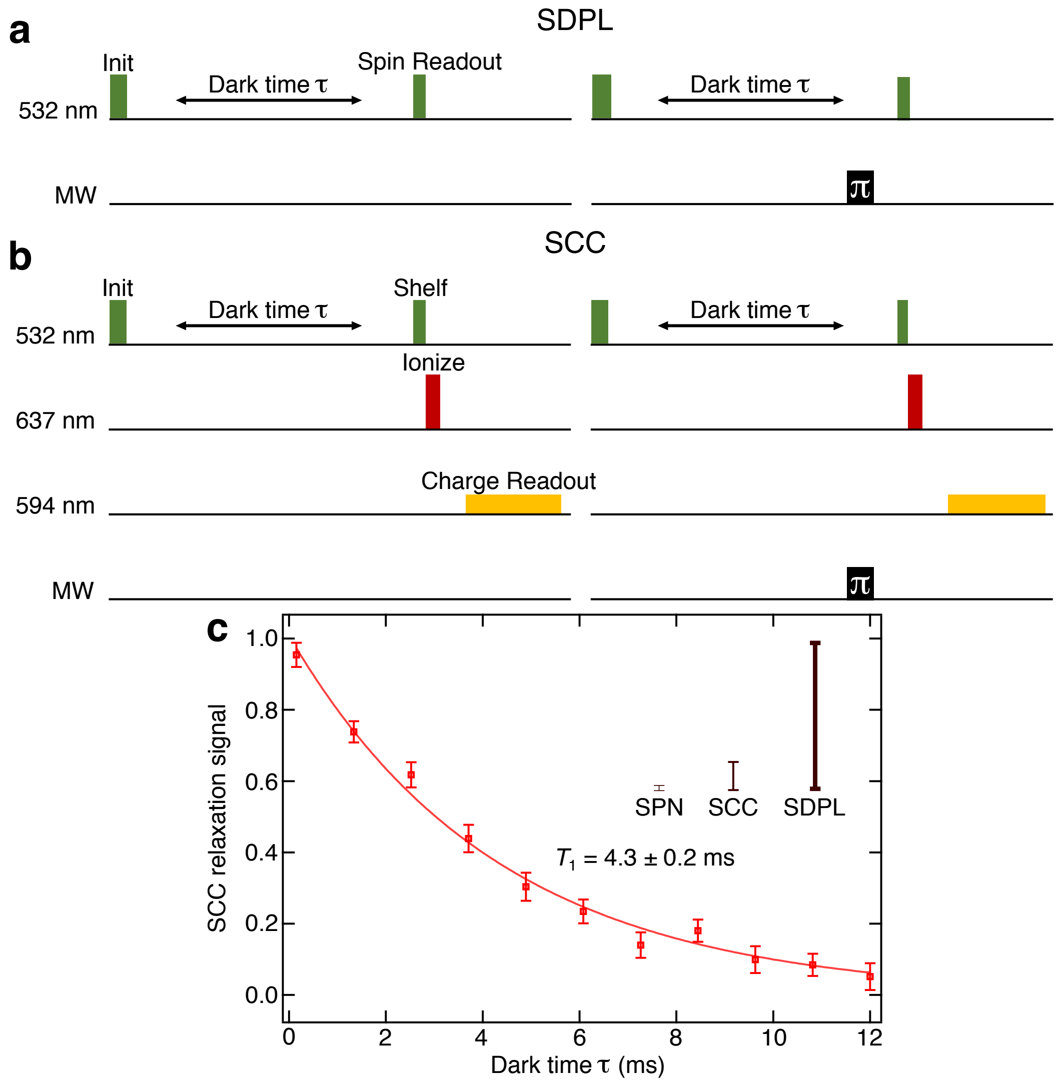

Supplementary Figure 3: Spin-to-charge conversion (SCC) T_1 measurement on a shallow NV center. **a** Conventional, spin-dependent photoluminescence (SDPL) T_1 measurement scheme. The π pulse swaps the $|0\rangle$ and $|-1\rangle$ populations immediately before readout, as explained in Supplementary Eq. 21. **b** Same preparation sequence as **a** but readout uses SCC. To convert spin to charge, we do a $\sim$ 300-μW, 60-ns green pulse to shelve $|\pm1\rangle$ into the singlet, immediately followed by a $\sim$ 30-mW, 40-ns red pulse in order to discriminately ionize $|0\rangle$ without touching the singlet population. We then perform charge-state readout with a $\sim$ 3-μW, 500-μs yellow pulse which discriminately excites NV$^-$. **c** SCC T_1 measurement on a shallow NV center. The measurement took 30 minutes. Plotted error bars represent the measured standard error, and are 5x larger than the spin projection noise (SPN) limit. The inset shows the relative magnitude of typical SPN, SCC, and SDPL error (1, 5, 25). Nominally, to achieve the same error the SCC measurement is 25x faster than SDPL.

More carefully accounting for the 3-level system yields a 5% smaller SPN limit.

At saturation laser powers of $\sim$ 1 mW at 532 nm, we collect $\sim$ 500 kCounts s^{-1} from a single NV. In this work, we typically operate at $\sim$ 300 μW with $\sim$ 180 kCounts s^{-1}. Under 594 nm laser excitation (used for spin-to-charge readout), we typically measure $\sim$ 5 kCounts s^{-1} for 3 μW incident power. In Fig. 3c of the main text and in Supplementary Figure 3 we experimentally measure $\sigma_{\mathrm{SCC}} \approx 5/\sqrt{2N} = 5\,\sigma_{\mathrm{SPN}}$. For spin-dependent photoluminescence (SDPL) readout, we typically measure $\sigma_{\mathrm{SDPL}} = 25\,\sigma_{\mathrm{SPN}} = 5\,\sigma_{\mathrm{SCC}}$, which is consistent with photon shot noise for the experimental parameters of a 400-ns long readout, $|0\rangle$-state PL of 180 kCounts s^{-1}, and PL contrast of 30% between $|0\rangle$ and $|\pm1\rangle$ states. Further, note that the SPN value quoted above assumes perfect initial polarization into NV$^-$ and $|m_s = 0\rangle$; in practice imperfect spin and charge polarization increases our experimental error by a factor of $\sim$ 1.3. Thus, in reality our measured readout error $\sigma_{\mathrm{SCC}} \approx 4\,\sigma_{\mathrm{SPN}}$.

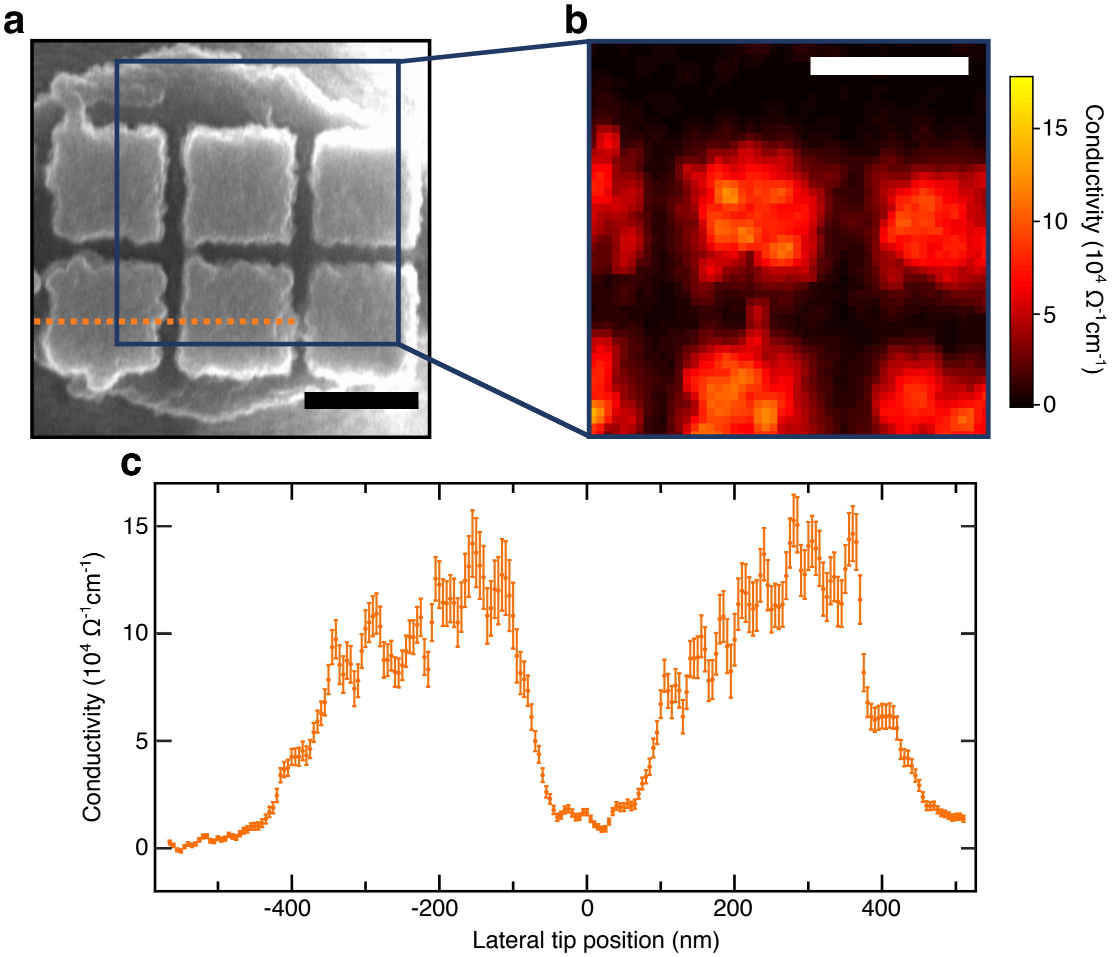

Supplementary Figure 4: Nanoscale conductivity imaging. The data in **b-c** shows the data in Fig. 3**b-c** of the main text, with T_1 converted to conductivity. **a** Scanning electron microscopy image of an Al nanopattern deposited onto an AFM tip, as depicted in Fig. 1a of the main text. **b** NV-based conductivity image of the area depicted by the 1 μm^2 blue square in **a**, produced by scanning the NV center over this area at a height (d_0) of 40 nm and with 20 nm pixel spacing. The height of 40 nm is extracted from a simulation fit with the model described in Supplementary Note 4.The conductivity is then calculated by using Eq. 3 of the main text with $t_{\text{film}} = 85$ nm. **c** High-resolution conductivity line scan of the dotted orange line in **a**, using spin-to-charge readout. More data points at the left edge of the scan are included in this plot than in Fig. 3c of the main text to show the conductivity decay to 0 (not included in the main text to keep the T_1 range below 3.5 ms to focus on the conducting regions). A simulation fit here also extracts a height of 40 nm and the conductivity is calculated in the same way as in **b**. The intrinsic T_1 is 6 ms for both NVs. Scale bars are 400 nm. Error-weighted, light smoothing is applied to the data in **b** and **c**, for which nearest neighbors receive an additional weight reduction by a factor of 2.5. Error bars are calculated by propagating the measured standard error of the photoluminescence for the single-τ measurement of T_1, and then propagating the error as prescribed by Eq. 3 of the main text. The extracted height of 40 nm from the simulation fit is approximate to $\pm$ 5 nm, which would then lead to a systematic offset of the plotted conductivity to $\pm$ 10%.

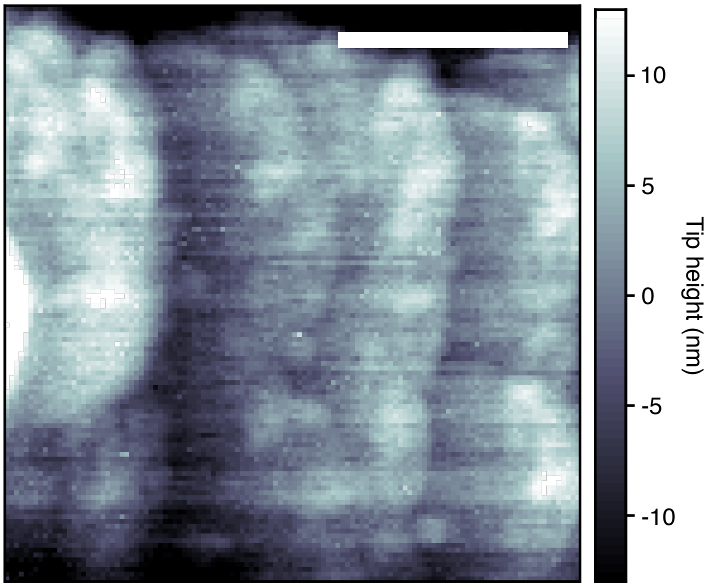

Supplementary Figure 5: AFM scan of the 1 μm^2 region imaged in Fig. 3b of the main text, taken immediately after the completion of the T_1 scan. The topographic features produced by the convolution of the 400-nm diameter diamond pillar and the nanopatterned sample do not correlate with the T_1 features in Fig. 3b, indicating that the T_1 features are not produced by a changing NV-sample separation d_0. The dark regions at the top and bottom of the AFM scan correspond to the edges of the nanopatterned sample. Neglecting these regions yields a root-mean-square variation of 5.1 nm over the entire region. For an average NV-sample separation of 40 nm these 5 nm variations correspond to 10% variations in the measured conductivity (an uncertainty of about 10^4 Ω^{-1}cm^{-1} at maximum points of conductivity), which is the approximate magnitude of the measured error in Supplementary Figure 4. Positive values of height correspond to retracting the nanopattern away from the diamond. Scale bar is 400 nm.

SUPPLEMENTARY REFERENCES

[1] C. Henkel, S. Pötting, and M. Wilkens, "Loss and heating of particles in small and noisy traps," Applied Physics B **69**, 379–387 (1999).

[2] Luke S. Langsjoen, Amrit Poudel, Maxim G. Vavilov, and Robert Joynt, "Electromagnetic fluctuations near thin metallic films," Physical Review B **89**, 115401 (2014).

[3] S Kolkowitz, A Safira, A A High, R C Devlin, S Choi, Q P Unterreithmeier, D Patterson, A S Zibrov, V E Manucharyan, H Park, and M D Lukin, "Quantum electronics. Probing Johnson noise and ballistic transport in normal metals with a single-spin qubit." Science (New York, N.Y.) **347**, 1129–32 (2015).

[4] B.A. Myers, A. Ariyaratne, and A.C. Bleszynski Jayich, "Double-Quantum Spin-Relaxation Limits to Coherence of Near-Surface Nitrogen-Vacancy Centers," Physical Review Letters **118**, 197201 (2017).

[5] B J Shields, Q P Unterreithmeier, N P de Leon, H Park, and M D Lukin, "Efficient Readout of a Single Spin State in Diamond via Spin-to-Charge Conversion," Physical Review Letters **114** (2015).

[6] C. Henkel, "Magnetostatic field noise near metallic surfaces," The European Physical Journal D **35**, 59–67 (2005).

[7] O. M. Bulashenko, O. V. Kochelap, and V. A. Kochelap, "Size effect on current fluctuations in thin metal films: Monte Carlo approach," Physical Review B **45**, 14308–14314 (1992).

[8] Manuel Guizar-Sicairos, Samuel T. Thurman, and James R. Fienup, "Efficient subpixel image registration algorithms," Optics Letters **33**, 156 (2008).

[9] M. Pelliccione, B.A. Myers, L.M.A. Pascal, A. Das, and A.C. Bleszynski Jayich, "Two-Dimensional Nanoscale Imaging of Gadolinium Spins via Scanning Probe Relaxometry with a Single Spin in Diamond," Physical Review Applied **2**, 054014 (2014).

[10] Niels Møller Israelsen, Shailesh Kumar, Mahmoud Tawfieq, Jonas Schou Neergaard-Nielsen, Alexander Huck, and Ulrik Lund Andersen, "Increasing the photon collection rate from a single NV center with a silver mirror," Journal of Optics **16**, 114017 (2014).
